# The stories of the ageing population in Luton, United Kingdom on their experience of periodontal diseases healthcare

**DOI:** 10.3389/fpubh.2025.1710973

**Published:** 2026-03-11

**Authors:** Karuna Preethi Velpula, Enemona Jacob

**Affiliations:** 1 School of Life and Medical Sciences, University of Hertfordshire, Hatfield, United Kingdom; 2 University of Hertfordshire, Hatfield, United Kingdom

**Keywords:** oral health, periodontal disease, ageing, Indian adults, NHS dentistry, cultural belief, Luton, health literacy

## Abstract

**Background:**

Oral health is an important part of general wellbeing, but in the United Kingdom there are substantive inequalities, especially based on ethnic minority and ageing. There is a poor quality of life, a lack of awareness and preventive measures, and periodontal disease contributes to loss of teeth, poor nutrition, and poor quality of life. Ageing adults among Indians in Luton are an under-studied group whose experiences could inform the interface of cultural, structural, and psychosocial determinants of oral health.

**Aim:**

The research aimed to understand the lived experiences of ageing Indian adults in Luton with respect to periodontal health, dental service provider access, and the impacts of cultural, familial, and emotional factors on oral health behaviours.

**Methods:**

A qualitative research design was adopted where semi-structured interviews of 10 ageing Indian adults living in Luton were conducted. Data were analyzed in terms of their themes using theoretical frameworks of the Health Belief Model, the Social Cognitive Theory, and Intersectionality to identify patterns and meaning in participant stories.

**Results:**

The results showed that the level of knowledge about periodontal disease was low; and most of the participants believed that the gum issues were not a big problem and that everyone got gum problems with ageing. The obstacles to care access were that the NHS was too expensive, too long, and too complicated; the quality of care in the private sector was too high, and it was unaffordable. Fear, mistrust, shame (psychology) also discouraged the use of dental services.

**Conclusion and recommendations:**

This study finds that the interaction of culture beliefs, systemic barriers, and psychosocial variables affects the oral health of older Indian adults in Luton. Periodontal treatment tends to be reactive and symptomatic with little preventive treatment. This research suggests that the main issues will be culturally sensitive oral healthcare education, family- and community-intervention, affordability, access and cultural competence of NHS dental care. It contributes new qualitative evidence on the need for culturally competent, family-engaged, and accessible dental care for ethnic minority older adult in the UK.

## 1 Introduction

One of the most common non-communicable oral diseases in the world is periodontal disease. The Global Burden of Disease data show that severe periodontitis is the sixth most common disorder in the world (1). Such a worldwide burden is aggravated by demographic changes. Research reports show that about two out of every three adults over 55 years’ experience periodontal disease (2). Although preserving natural teeth in old age is a positive phenomenon, it also exposes the population to the pathology of periodontal diseases (3). This tendency highlights the shift in demand for long-term periodontal care, which should consider competing frailty and comorbidities in advanced age groups (3). Its high rate in big parts of society suggests that continued monitoring and interventions must be continued.

Substantial evidence indicates the systemic implications of periodontal disease. Chronic periodontitis induces the inflammatory process by releasing cytokines like C-reactive protein and interleukins, which can negatively impact vascular health (4). Meta-analyses indicate that periodontitis patients are older adults who have a high risk of developing cardiovascular diseases. Similarly, diabetes proves to have a bidirectional correlation with periodontal disease. Chronic inflammation in the periodontal region negatively affects glycemic control, and an increase in periodontal care results in a decreased level of HbA1c (5). Additional studies indicate that periodontal disease may have a connection with cognitive deterioration, but the association is not yet conclusive (6). These relationships indicate the multidimensional interdependence of oral and overall health in older age.

There are complex obstacles facing older adults that prevent access to dental care. A UK scoping study indicated the major barriers as cost, mobility limitations, dental fear, and lack of availability of services (7). Such obstacles are exacerbated by such passive barriers as low perceived need in the wearers of dentures (7). Socio-economic deprivation and ethnic minority status are also social determinants, which contribute to even more challenges (8). The survey by The Guardian also indicated that poorer people are disadvantaged in accessing NHS dental and GP services. Also, there are still shortages in the workforce and structural service constraints. These results suggest that low oral performance among older people is not entirely a clinical necessity but a systemic, societal, and personal barrier.

Domiciliary dental care is an important alternative for individuals who cannot visit the traditional clinic because of frailty or mobility issues. Domiciliary services have been proven to be safe, effective, and appreciated by care-dependent adults (9). Nevertheless, there are still huge access-related obstacles concerning providers’ availability, price, and coordination of services. Policy frameworks that provide domiciliary guidelines are still divided and poorly funded. Such facilitators are caregiver training, inter-professional coordination, and supportive commissioning (9). The development of domiciliary programs implies investment in the skills of the workforce and contractual incentives. In the end, domiciliary care can solve life-threatening access problems, yet it is underutilised due to systemic neglect.

The management of periodontal disease in the older adult population needs combined policies of public health that address epidemiological, systemic, and access aspects. Although population-based interventions like fluoridation provide minimal benefits, they are not effective in reaching out to marginalised older populations (8). The policy should encourage including oral health in the chronic disease pathways to exploit systemic health connections (10). Outreach, workforce training, and service delivery models are necessary on a culturally and socioeconomically informed basis. Specifically, domiciliary dental services should be structured, commissioned, and funded to scale (9). Further surveillance and policy adjustments will guarantee that older adult-oriented oral health services will accompany the increasing population of older adult.

The United Kingdom has realised that oral health is important in its public health policies. Nevertheless, current models fail to meet the special needs of older ethnic minorities. To illustrate, the NICE Guideline NG48 states that oral care in care homes is necessary and fails to provide advice on cultural adaptation and specific outreach. The local data of Luton, where more than 12.5 percent of the population aged 55 or older has Indian origins, indicate that older Indian adults tend to feel underserved (11). Such people face structural barriers like language challenges and lack of culturally relevant communication in dental services. The result is that this population group is less likely to use NHS dental services.

Structural inequalities among older ethnic minorities also increase these disparities. The local NHS dental uptake data indicate that people over 55 in Luton use dental services about 30 percent less than the national rates (12). This discrepancy indicates the general infrastructural limitations, including the lack of NHS providers in the areas of deprivation, longer wait times to make appointments and difficulties with transportation among older people who are less mobile (7). Moreover, socio-economic forces facilitate oral disease despite the presence of services (13). Such structural elements indicate that current NHS structures mostly fail this group of older adults.

Ethnicity is a crucial factor in the development of diseases as well as participation in health care. A study in East London showed that South Asian adults, specifically Indian people, experience more periodontal pocketing than White British people, despite the consideration of socio-economic status (13, 14). Moreover, they are more likely to preserve their teeth and have a high level of periodontal problems because of the insufficient use of aggressive treatment methods (13). The reasons behind this tendency are cultural practices, oral hygiene habits, anxiety about dental procedures, and low trust in the health system (14). This combination of biological, cultural, and social influences gives this group an aggravated risk profile.

The study core purpose was to explore the experiences of the ageing Indian population in Luton regarding periodontal disease healthcare.

Based on the study aim, the following objectives were to be achieved

- To explore the lived experiences and perceptions of periodontal health among ageing Indian adults in Luton, with particular attention to how they interpret oral health changes in the context of ageing.
- To examine the structural and systemic barriers faced by ageing Indian adults in Luton when engaging with dental services, and to analyse how these challenges influence their decisions to seek or delay care.
- To investigate the role of cultural traditions, family relationships, and community norms in shaping the oral health behaviours and decision-making of ageing Indian adults in Luton.

## 2 Methods

### 2.1 Study design

The study aim was to explore the experiences of the ageing Indian population in Luton regarding periodontal disease healthcare. Semi-structured qualitative interview approach was used because it is a flexible and deep method (15). They strike a balance between the guidance of the researcher and the conversational flow, thereby enabling the participants to expound on the relevant issues and, at the same time, cover the important areas.

### 2.2 Participant selection

The target population in this research includes Indian adults aged 55 + who live in Luton. These people can be at different stages of interaction with periodontal health services. The lower age boundary of 55 was selected to reflect early onset of oral health disparities that may appear before retirement age. Epidemiological evidence suggests that periodontal conditions often progress significantly in adults from their mid-50s, particularly among ethnic minority groups with long-term exposure to social and structural inequalities in healthcare access. The aim is to seek breadth in lived experience as opposed to prevalence. The available population consists of people who participate in community events, receive domiciliary services, or work with healthcare groups. This renders purposive sampling viable without violating diversity in living arrangements and lifestyles. It makes provision for inclusion based on factors like gender, language, income, and service engagement (Table 1).

**Table 1:** Inclusion and exclusion criteria for study participants.

Criteria type	Description
Inclusion criteria	Aged 55+, of Indian origin, resident in Luton, fluent in English/Hindi/Telugu, cognitively able to consent and participate, recent dental care contact (last 2 years)
Exclusion criteria	Severe cognitive impairment, terminal illness, and unable to communicate

The study’s selection of the population reflects diverse experiences in this community. It involves participants who access NHS dentistry, privately funded care, outreach in churches and domiciliary care. The purposive sampling approach enables the appearance of diversity and the retention of the core phenomenon of the study periodontal healthcare experience. Through community networks, participants will be invited to build trust and ensure the highest cultural accessibility.

The sample size in qualitative research is determined by the concept of saturation, whereby no new themes are identified after further interviewing (16). This approach is different from statistical power calculations, which are more appropriate for quantitative studies. Rather, saturation assures depth and richness of insight. Qualitative work on oral health with ethnic older adult literature indicates that the majority of studies achieve saturation at 10 to 20 interviews (16). Thus, 10 (6) interviews are reasonable to avoid repetitive experiences.

Purposive sampling was chosen because it allows for the purposeful inclusion of respondents who are most likely to offer relevant insight (17). The researcher created a flyer concerning the research which was used in the participant’s recruitment. The flyer was advertised across different social media sites such as Telegram, Instagram, and Facebook. Snowballing was also a useful approach since it allowed the researcher to reach participants that would not be reached with other approaches. The snowballing ensured recruitment of reliable participants and built trust with them. Stratification was done based on gender and living conditions, whether as an independent person, in a multi-generational household, or in care environments (Table 2).

**Table 2:** Recruitment summary by source.

Recruitment source	Number approached	Responded/expressed interest	Eligible after screening	Participated (interviews completed)	Refused/withdrew	Response rate (%)
Community events (temple, cultural associations)	18	10	8	6	4	55.6
Domiciliary services and healthcare groups	10	6	5	2	1	60.0
Social media (Telegram, Instagram, Facebook)	25	7	4	1	3	28.0
Snowball sampling (peer referrals)	8	5	4	1	1	62.5
Total	61	28	21	10	9	45.9

The most effective recruitment route was ‘domiciliary services and healthcare groups’ because it had a higher response rate.

### 2.3 Setting and data collection

The research was conducted in Luton, a town in southeast England where the ageing Indian community is high (11). The data collection was only conducted through teleconferencing platforms; the participants participated in online interviews. This provided them with flexibility. We scheduled an interview based on the participant’s availability and comfort.

A guide on the interview was developed; this was considered effective in collecting health data. It included open-ended questions that will target access, attitudes, and cultural influences, including: “Can you tell me about a situation when you made a decision to treat your gums?” The interview guide was flexible to follow the leads of the participants. This assists in the authenticity and capturing of meaningful data.

**Figure 1:**
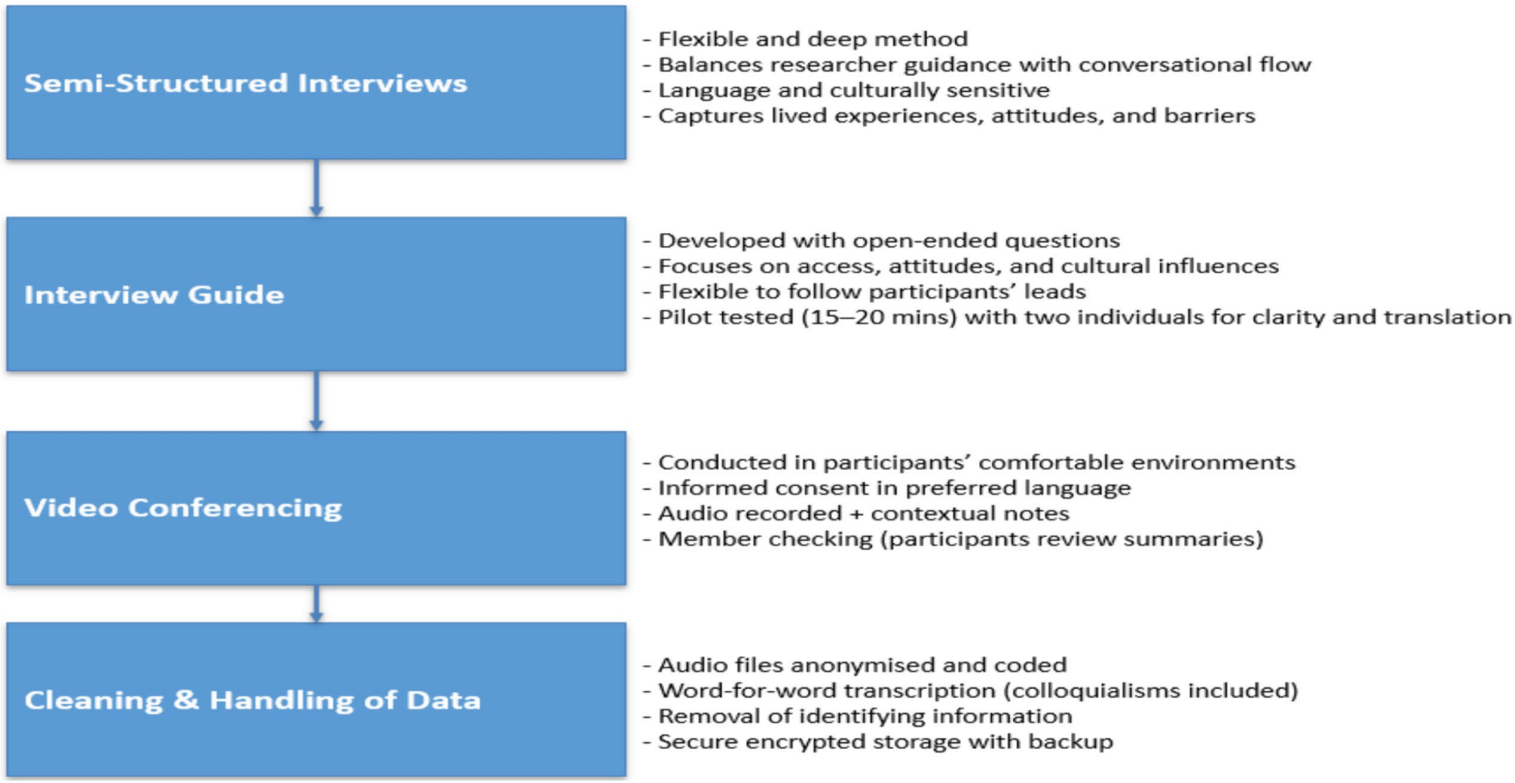
Study data collection process.

The flow, timing, and relevance of the guide will also be tested by interviewing two people who are not in the main sample as pilots. These pilots will take approximately 15–20 min and give feedback on the clarity of questions. The changes were made according to the participants’ responses and understanding. Piloting is also useful in perfecting translation and detecting ambiguous phrases (Figure 1).

### 2.4 Data analysis

**Figure 2:**
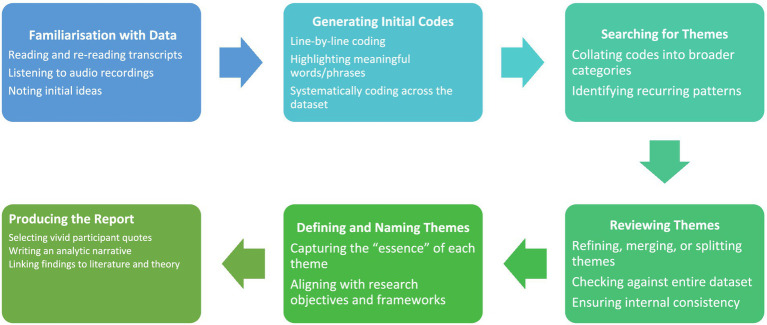
Data analysis process.

I adopted the six-step thematic analysis framework of Braun and Clarke to interpret the narratives of the ageing Indian adults in Luton (Figure 2).

Thematic analysis was applied to analyse data according to the six-step approach by Braun and Clarke (18). First, immersion and familiarisation were done by reading transcripts repeatedly. Line-by-line coding was performed on the basis of semantic meaning. A list of codes was developed based on the data that was collected and the research objectives. These codes were organised into some initial themes, which were revised and refined back and forth. This procedure aids in the interpretation of trends in regard to access, beliefs, and contextual influences. Themes were specified, labelled, and presented with quotes from the participants. Such an approach guarantees systematic and reflexive analysis in accordance with interpretive principles.

### 2.5 Ethical approval and participants protection

The research complied with the principles of Respect of Persons, Justice, Beneficence and Nonmaleficence. Institutional oversight was achieved by the ethical approval of the University Ethics Committee. Informed consent was sought both verbally before the interview and in written forms. The participants were made aware of their rights to withdraw without any penalty. Emotional health will also be observed, and the interviewers will pause or halt when the participants are distressed. These protocols guarantee the ethical integrity and safety of the participants during the study.

## 3 Results

Steps followed for my data analysis (Table 3).

**Table 3:** Steps followed for my data analysis based on thematic process by (18).

Thematic step	Examples from participants (Arjun → Kiran)	Initial themes
Familiarisation	Arjun: cavity treated after wife urged; Ravi: bleeding gums, fears cost; Meera: bleeding gums, trusts dentist; Anita: sensitive teeth, veneers, private care; Priya: occasional bleeding, neglects dentist; Sita: long NHS waits, gum pain; Lakshmi: gum bleeding, avoids dentist; Shobha: hesitates to seek care; Radha: avoids dentist due to cost; Kiran: persistent gum bleeding, reluctant care	Limited awareness, access barriers, family influence, emotional dimensions
Initial ideas	Common issues: bleeding gums, delays, neglect, reliance on family; Differentiation: some proactive (Anita, Meera), others neglectful (Priya, Radha)	Shows spectrum of awareness and coping
Generating codes	“Bleeding while brushing,” “appointments expensive,” “wife convinced me,” “painful treatment,” “private dentist,” “delay visits,” “fear of dentist”	Codes grouped into awareness gaps, financial/access barriers, family role, fear and emotional distress
Line-by-line coding	Highlighted participant phrases: Arjun: “wife compelled me”; Ravi: “dentists are expensive”; Meera: “dentist advice helped”; Anita: “I floss daily”; Priya: “not seen dentist in years”; Kiran: “gum bleeding continues”	Clear clustering around avoidance, cost, family influence, awareness
Searching for themes	Patterns: - Family as motivator (Arjun, Meera) - Cost/delay barriers (Ravi, Priya, Radha, Kiran) - Emotional reluctance/fear (Shobha, Lakshmi) - Higher awareness in private care (Anita)	Emerging themes consistent with report
Reviewing themes	Checked across all 10 interviews → overlap in *barriers*, *limited awareness*, *family role*, *fear*. Some unique: Anita (private dentistry), Kiran (persistent but untreated symptoms)	Ensures themes capture diversity but remain coherent
Defining and naming themes	“Limited Awareness” = normalising bleeding as ageing (Priya, Radha) “Access Barriers” = NHS wait times, high cost (Ravi, Kiran) “Family Influence” = spouse encourages care (Arjun, Meera) “Cultural Beliefs” = reliance on remedies, shame in seeking care “NHS vs. Private” = Anita’s experience “Emotional Dimensions” = fear, avoidance (Lakshmi, Shobha)	Themes aligned with objectives
Producing report	Selected vivid quotes: Arjun: “my wife compelled me…” Ravi: “dentists are expensive” Priya: “not seen dentist for years” Anita: “I floss daily” Kiran: “gum bleeding continues”	Used as evidence in analytic narrative

All the names used in the table and in the interview, transcripts are pseudo names.

### 3.1 Key findings

The collected data was analyzed using thematic analysis to understand the emerging themes. The table below summarizes the themes found in the collected data (Table 4).

**Table 4:** Themes in the data.

Theme	How the theme emerged from transcripts	Supporting evidence (quotes/transcript extracts)	Analytical interpretation
Limited awareness of periodontal health	Participants often described bleeding gums and sensitivity as ‘part of ageing’ rather than preventable disease, showing limited awareness of periodontal health. Quotes included: ‘I do not know much about gum disease… I just brush twice daily’ and ‘They think it’s a small thing.’	‘I do not know much about gum disease… I just brush twice daily.’; ‘They think it’s a small thing.’	Low awareness normalises gum disease as part of ageing, reducing motivation for preventive care.
Barriers to accessing dental care	Most respondents described NHS dental care as difficult to access due to long waiting times and high costs. Quotes included: ‘Getting an NHS appointment is really hard’ and ‘Even just the consultation is £90, too expensive.’	‘Getting an NHS appointment is really hard.’; ‘Even just the consultation is £90, too expensive.’	Systemic barriers like cost, waiting times, and appointment difficulties delay or prevent treatment-seeking.
Influence of cultural beliefs and practices	Several participants mentioned using turmeric, clove oil, or salt rinses as remedies, delaying professional care. One said: ‘They used to tell like some homemade tips, that is very helpful actually.’	‘They used to tell like some homemade tips, that is very helpful actually.’; ‘Using turmeric and lemon.’	Cultural remedies fill gaps left by systemic barriers but can perpetuate delays in professional care.
Family influence	Decisions about seeking care were often shaped by family encouragement or neglect. For example: ‘My wife convinced me to see the dentist’ and ‘My husband books for both of us.’	‘My wife convinced me to see the dentist.’; ‘My husband books for both of us.’	Family acts as both a barrier and facilitator in oral health behaviour, highlighting relational influence.
NHS versus private dentistry	Participants compared NHS with private care, describing NHS as overburdened, while private practices were ‘cleaner, faster, and more modern’ but unaffordable. Example: ‘Private ones have better equipment but are too costly.’	‘Private ones have better equipment but are too costly.’; ‘NHS dentists are rundown and busy.’	Perceptions of unequal quality between NHS and private dentistry highlight inequities in access.
Psychological and emotional dimensions	Emotional responses such as fear, shame, and embarrassment discouraged visits. Quotes included: ‘I feel quite scared of dentists’ and ‘It’s very painful, so I avoid going until I must.’	‘I feel quite scared of dentists.’; ‘It’s very painful, so I avoid going until I must.’	Fear and embarrassment compound structural barriers, leading to avoidance and delayed treatment.

#### 3.1.1 Limited awareness

Participants exhibited low levels of awareness of the fact that periodontal disease is a preventable condition and tended to attribute such symptoms as gum bleeding or sensitivity to ageing. This observation is consistent with other studies that show that low oral health literacy is still present in older South Asian populations in the UK (19). Most participants failed to associate the early symptoms with underlying pathology, and instead, they accepted them as part of their ageing. This normalisation is a blow to preventive care like frequent scaling, flossing, or early referral to a specialist. The inability to see oneself as prone to illness lowers the chances of behavioural change, as stated by the Health Belief Model (20). Where information existed, it was usually incomplete and was based on family or community sources, but not on health promotion campaigns.

There was a lack of understanding of preventive dentistry among participants, with oral health maintenance being defined mainly in the context of brushing frequency as opposed to an overall approach. Only a few people cited interdental cleaning or the awareness of periodontal pocketing as signs of progression of the disease. Instead, people talked about reactive care, i.e., trying to get treatment only when the pain or other visible symptoms become unbearable. Such a reactive orientation aligns with the results of studies of South Asian populations who claim that they visit dentists only in case of emergencies (14). The inability to be preventatively oriented is compounded by the fact that there are no culturally specific educational tools in Luton.

#### 3.1.2 Access barriers

The most common theme in all interviews was the inability to access cheap and timely dental care. The main reason given by participants was financial limitations, especially in relation to the NHS charges that were felt to be unpredictable and costly. Some participants said that they delayed treatment due to the fear of the possibility of hidden charges, which has been reported in UK dentistry (7). NHS exemptions were not always clearly understood, and eligibility criteria were considered to be obscure. These results indicate that obstacles are not only financial but also informational, which strengthens the existing disparities. Structural disparities in service delivery also exacerbated cost-related issues, with participants explaining that they had difficulty obtaining appointments in a timely manner.

Language and cultural barriers also contributed to the fact that they were deterred. Some of the respondents complained about feeling discomfort when interacting with dental offices, where they felt that the communication was hurried or culturally insensitive. The use of family members to translate also posed a challenge to those with low levels of English proficiency because it led to privacy issues and a lack of autonomy when making healthcare decisions. Structural obstacles such as an inaccessible appointment system and inflexible scheduling also worsened the case of older participants who were limited in mobility. The compounding effect of financial, linguistic and organisational barriers depicts how intersectional disadvantages limit the utilisation of services.

#### 3.1.3 Cultural beliefs

Cultural beliefs and practices were identified to affect how the participants perceived and responded to the periodontal symptoms. Some of them claim to use natural remedies such as clove oil, turmeric, or salt rinses to cure the pain or tenderness of the gums. These practices, however, only provided temporary relief of the situation, which slowed down the access to professional services, thus increasing the pace of the disease development. The employment of home remedies is an indicator of intergenerational knowledge systems based on Indian cultural traditions of Ayurveda and home-based care. Although these practices may not be necessarily harmful, they are dominant in the absence of awareness of preventive dentistry, thus resulting in underutilisation of formal services.

#### 3.1.4 Family influence

Family structures were very instrumental in influencing the oral health behaviours and access to healthcare among the participants. Women especially reported that oral health decision-making was dependent upon the approval or encouragement of the spouse, and this was indicative of gendered relationships in the South Asian household. Caregiving obligations caused some women to postpone dental appointments because they had to prioritise the needs of their families over their own health. These testimonies are consistent with larger gendered health inequities, in which women have limited health-seeking behaviour due to cultural demands of responsibility and decency (14). On the other hand, other participants described positive family engagement, including spouses urging them to visit on a regular basis or children making appointments.

#### 3.1.5 NHS vs. private care

Participants had varied experiences of NHS dentistry, and cost-effectiveness was cited as its main strength. However, there was a general dissatisfaction with NHS services, especially with regard to the availability of appointments and the perceived lack of personalised care. Numerous participants reported hurried consultations in which they did not feel that their concerns were properly addressed. Participants were more likely to contrast these experiences with the notion that private dentistry is more attentive and consistent. It was also believed that private treatment was unaffordable to most people, and this led to the lower-income households feeling left out. The superiority of private care created a sense of frustration and mistrust of the NHS dentistry. Some participants were sceptical that NHS dentists were more financially efficient than patient-centred, which created the feeling of neglect. In comparison, the private practitioners were said to be more communicative and culturally sensitive, although such services were mostly not accessible.

Findings reveal that perceptions of NHS dentistry as rushed, impersonal, and inaccessible compared with private care undermine trust and equity in service provision. Participants’ experiences of long waits, limited communication, and cultural insensitivity point to systemic under-resourcing rather than individual choice. Policy change is needed to strengthen NHS dental capacity through improved funding, workforce allocation, and culturally competent training (2). Integrating interpreter services and patient-centred communication protocols could enhance accessibility and satisfaction. Addressing these structural gaps would promote fairness, rebuild trust, and ensure that quality oral healthcare is not dependent on income or cultural familiarity.

#### 3.1.6 Emotional dimensions

Fear and mistrust were key emotional responses to dental care that were reflected in the narratives of participants, and were major drivers of behaviour. Dental fear was usually based on previous unpleasant experiences, whether they were painful procedures or unprofessional communication with the practitioners. Such memories led to long-term avoidance behaviours that perpetuated the use of symptomatic or home-based care. The respondents reported fear of physical pain during treatment as well as the financial cost. The combination of emotional and structural barriers in this way created cumulative barriers to care-seeking.

Shame also appeared to be a big emotional obstacle. Others stated that they were embarrassed by their teeth or gums and feared being judged by the dentist. This sense of stigma discouraged frequent visits, and this led to avoidance patterns. The shame was especially strong among women, who referred to oral health as a manifestation of personal hygiene and self-care. These accounts demonstrate the importance of cultural beliefs of humility and self-representation to medical experiences. Shame is an emotional burden that is not commonly discussed in oral health interventions, which tend to be limited in scope to structural or behavioural obstacles.

## 4 Discussion

The research established that the participants had limited knowledge about periodontal disease beyond the visible or symptomatic signs of the disease, like gum bleeding or pain. Most people explained these symptoms as part of the normal ageing process instead of conditions that could be prevented through clinical intervention. Such perception is indicative of a larger trend of poor oral health literacy among South Asian communities in the UK (13). Preventive measures like interdental cleaning, routine scaling or periodontal pocket monitoring were not frequently mentioned by the participants. Rather, the dental visits would be delayed until the pain became unbearable, further instilling a reactive versus preventive approach. This is in line with the Health Belief Model, which argues that low perceived susceptibility and perceived benefit decrease the chances of preventive health behaviour (20). Poor awareness is thus a major impediment to early diagnosis and treatment of periodontal disease among minority ageing groups.

The other aspect of the lack of awareness was the information sources. Participants tended to use informal knowledge that was shared through family, community or cultural networks instead of evidence-based health information. Although these networks are valuable support systems, they can, in fact, perpetuate misconceptions or fatalistic beliefs about oral health. As an example, the participants explained that gum bleeding is normal or part of growing old, which deterred them from seeking early treatment. Valdez et al. (19) note that ethnic minority groups tend to have disparities in access to culturally appropriate oral health information, which is why disparities persist. This is aggravated by the lack of specific public health campaigns for the older South Asians in Luton. Thus, to improve the periodontal health outcomes, it is necessary not only to increase the awareness on an individual level but also to create system-level interventions that would offer culturally appropriate oral health education.

The cost factor was always mentioned as a major impediment to dental care. Respondents reported that NHS charges were heavy and unpredictable, thus causing delays in treatment until conditions became serious. This is in line with the findings at the national level that affordability is one of the greatest obstacles to oral healthcare in the UK (7). The economic burden is also disproportionately borne by lower-income households, which are over-represented among ethnic minorities because of structural disparities in the labour market and income (21). In this sense, the economic aspect of access cannot be isolated from the other social factors of health. Unless the NHS dental pricing and subsidy schemes are reformed, cost will continue to be a constant obstacle to ageing minority adults.

Such financial issues were compounded by structural and organisational barriers. Respondents complained about the inability to get an appointment in the NHS, long waiting lists, and short consultations. NHS Digital (12) has also reported the reduction in NHS dental capacity, especially after the COVID-19 outbreak, and unmet needs in England. The problem was exacerbated by the inability to communicate and the absence of culturally sensitive interpretation services among the participants with poor English language skills. A study by (22) supports this claim and shows that linguistic and cultural barriers also marginalise minority patients, adding to mistrust and underutilisation. These results depict how financial, organisational and cultural barriers interact, forming a multilayered disadvantage to ageing Indian adults in Luton. These obstacles must be overcome by changing the system to comprehend the complexity of oral health disparities.

The cultural beliefs had a significant impact on the way the participants conceptualised and reacted to the issue of periodontal health problems. Clove oil, turmeric, or salt rinses were some of the traditional remedies that could be used to treat gum pain or sensitivity as a first-line treatment. These remedies are founded on Indian Ayurvedic traditions, which ensure the continuity and resourcefulness of migrant communities. Nonetheless, the use of such remedies also tended to slow professional intervention and, as a result, the development of periodontal disease. According to (14), South Asians in the UK are more likely to retain their natural teeth than the White British, but they are more likely to have untreated periodontal disease. These results indicate that cultural practices need not be rejected but incorporated into learning strategies that can help close the gap between traditional beliefs and biomedical knowledge. The appreciation of cultural practices can be used to build trust and enhance the interaction with formal services.

Fatalism was another theme that reoccurred in the stories of the participants. The attitude of many was that tooth loss and gum disease were part and parcel of ageing, and this discouraged the desire to adopt preventive measures. This inevitability is consistent with the studies that have demonstrated fatalistic beliefs towards chronic illness in the South Asian population. Fatalism may be viewed as a cultural conviction as well as a coping mechanism in situations of structural exclusion, where people do not feel that they have access to services.

The family dynamics were also important in the way the participants engaged in oral health behaviours and access to care. The women in the study explained that they would put the health of spouses and children before their own and would postpone dental visits until they had time to attend to their family. This can be compared to the studies that discovered that women of South Asian descent tend to put their families first before their health. The approval of spouses was also cited as a factor that made women seek care or not, which shows that oral health decision-making was not completely independent. These results show that oral health behaviours are entrenched in the household dynamics where the overall household priorities supersede the health-seeking behaviour of an individual.

Respondents were not satisfied with NHS dental services and frequently reported rushed visits, excessive wait times, and the feeling that they did not receive personal care. These grievances are associated with the countrywide statistics that the NHS dentistry is overburdened, and the reports about the unmet demand and the disparity of services are broad (12). People did not trust the service, and the notion that NHS care was all about efficiency and not about the well-being of patients was a common theme. Participants who had undergone NHS dentistry felt that NHS dentists were financially motivated to offer the least care possible, which furthered a feeling of neglect. Such perception, whether true or false, erodes patient trust and prevents the use of preventive services. The opposite was true when it comes to private dentistry, which was always reported to be more attentive, personalised and culturally sensitive. Participants felt that the private practitioners were more open to explaining the processes and discussing issues. The affordability was, however, a key obstacle, especially to the lower-income participants.

Psychological factors have been found to be strong determinants of participants’ oral health behaviours. The fear of dental treatment was widespread, and it was usually based on the previous traumatic experiences of painful extractions or insensitive communication of practitioners. Dental fear has been well documented as a significant impediment to service utilisation, especially in the older adult population (23). Fear was also compounded by mistrust in NHS dentistry in this study, where the participants felt that their concerns were not addressed fully. Such psychological obstacles gave way to avoidance patterns, which further strengthened the use of crisis-based care and traditional solutions. Remarkably, fear was associated not only with physical pain but also with financial anxiety, which is a sign of the twofold burden of psychological and structural impediments.

### 4.1 Critical analysis of themes in relation to theoretical frameworks

The study’s themes collectively illustrate the multidimensional nature of oral health behaviour among older Indian adults in Luton, where individual beliefs, social contexts, and intersecting inequalities interact to shape care-seeking patterns. The Health Belief Model (HBM) helps explain the limited awareness and reactive orientation observed among participants (7). Low perceived susceptibility—evident in viewing bleeding gums as part of ageing—reduces preventive motivation. However, HBM’s focus on individual cognition neglects the structural and cultural constraints revealed here, such as financial barriers, linguistic exclusion, and institutional mistrust. These findings confirm critiques that HBM underrepresents systemic and emotional influences, including fear and shame, which override rational risk–benefit reasoning in this group.

The Social Cognitive Theory (SCT) provides further insight into the role of environmental and relational factors. Family encouragement and peer norms strongly shaped participants’ health actions, aligning with SCT’s emphasis on observational learning and self-efficacy. Yet, structural limitations—scarce NHS appointments, inflexible systems, and socioeconomic disadvantage—restricted participants’ ability to act on these social reinforcements. This demonstrates SCT’s partial limitation: while it recognises social influence, it assumes a level of agency not always possible within constrained environments.

The Intersectionality framework is essential for understanding how ethnicity, age, gender, and class intersect to produce compounded disadvantage. Cultural practices such as using home remedies reflect both resilience and marginalisation, arising from linguistic isolation and limited culturally appropriate information (7). Women’s narratives of caregiving and modesty reveal gendered inequalities in oral health prioritisation, while the contrast between NHS and private care underscores class-based exclusion. Intersectionality therefore situates individual beliefs and behaviours within broader systems of inequality that neither HBM nor SCT fully capture. Together, these frameworks highlight that improving periodontal health among ethnic older adult requires not only behavioural change but structural reform and culturally responsive service design.

### 4.2 Limitations

The small, non-representative sample of 10 ageing Indian adults living in Luton is a limitation of this study as it limits the scope of generalizability of the findings to the general South Asian or minority groups in the United Kingdom. The study was a qualitative inquiry, although depth, not breadth, was desired, the small sample could have missed significant differences of experiences among the socio-economic or linguistic or religious subgroups of the Indian community. In addition, it is possible that due to the use of self-reported accounts, there is a risk of recall bias and social desirability bias that could have affected the description of oral health behaviours by the participants. Pragmatism would also have restricted the development of rapport, and would have disqualified people who were not digitally savvy or had no access to the interview due to the use of online interviews or scheduled interview.

## 5 Conclusion

Poor health literacy and fatalistic beliefs limit the preventive behaviours due to limited awareness of periodontal disease. Structural barriers limit access to care, including affordability, NHS capacity, and linguistic barriers. Cultural practices can be resilient and a source of continuity, but can also be a barrier to the use of biomedical care when used alone. Family influence is found to be both a limitation, in terms of gendered expectations, and an asset, in terms of intergenerational support. The emotional reactions like fear and shame contribute to avoidance, whereas the systemic inequalities between the NHS and private care increase the disparities. Collectively, these results describe the interplay of cultural, structural, and psychological determinants in the development of oral health outcomes.

Culturally and structurally specific public health practice is required to directly address cultural and structural barriers to improve periodontal health outcomes in ageing Indian adults. Policymakers should also increase the interaction between dental services and community organisations and integrate oral health into ageing and chronic disease policies. The policy shift to equity and inclusivity is possible through the placement of oral health within the broader determinants of health.

To address the disparities highlighted in participant experiences, three key policy recommendations are proposed

- Increase funding to reduce waiting times and support flexible appointment systems for older adults, including domiciliary and weekend services.
- Mandate regular staff training on cultural communication, use of interpreters, and sensitivity to South Asian oral health beliefs to improve patient trust and understanding.
- Partner with local Indian community centres and religious institutions to deliver multilingual oral health education, preventive check-up days, and guidance on navigating NHS entitlements, reducing reliance on informal remedies and ensuring equitable access to care.

Research on interventions is also required to assess the effectiveness of culturally adapted oral health education, family-centred approaches and community-based outreach. Community-based participatory research (CBPR) is one participatory research approach that might help in ensuring that affected communities are part of the design of interventions (24). Engaging communities in the research process will enable research studies to generate findings that are rigorous and socially effective.

## Data Availability

The original contributions presented in the study are included in the article/supplementary material, further inquiries can be directed to the corresponding author/s.
