# Correction: The stories of the ageing population in Luton, United Kingdom on their experience of periodontal diseases healthcare

**DOI:** 10.3389/fpubh.2026.1832834

**Published:** 2026-04-13

**Authors:** Karuna Preethi Velpula, Enemona Jacob

**Affiliations:** 1 School of Life and Medical Sciences, University of Hertfordshire, Hatfield, United Kingdom; 2 University of Hertfordshire, Hatfield, United Kingdom

**Keywords:** oral health, periodontal disease, ageing, Indian adults, NHS dentistry, cultural belief, Luton, health literacy

In the published article, there were errors in the affiliations and the correspondence.

The affiliation “School of Education, University of Hertfordshire, Hatfield, United Kingdom” was erroneously assigned to author Karuna Preethi Velpula. This author only has “School of Life and Medical Sciences, University of Hertfordshire, Hatfield, United Kingdom”.

Author Karuna Preethi Velpula was erroneously assigned as a corresponding author. The only corresponding author is Enemona Jacob.

The original version of this article has been updated.

